# Quantitative evaluation of viral interference among Egyptian isolates of highly pathogenic avian influenza viruses (H5N1 and H5N8) with the lentogenic and velogenic Newcastle disease virus genotype VII in specific pathogen-free embryonated chicken eggs model

**DOI:** 10.14202/vetworld.2019.1833-1839

**Published:** 2019-11-23

**Authors:** Mohamed A. Soliman, Ahmed A. Nour, Ahmed M. Erfan

**Affiliations:** National Laboratory for Veterinary Quality Control on Poultry Production, Animal Health Research Institute, Giza 12618, Egypt

**Keywords:** avian influenza virus, Newcastle disease virus, real-time polymerase chain reaction, viral interference

## Abstract

**Background and Aim:**

Mixed infections of the highly pathogenic avian influenza virus (HPAIV) and Newcastle disease virus (NDV) are considered the most distressing problem of the poultry industry. The problem arises due to the influence of a hidden virus on the replication of another suspected virus. Consequently, misdiagnosis of the real cause of disease may become a source of infection for other healthy stock by transmission and dissemination of the hidden virus. This study aimed to determine the impact of HPAIV and NDV on each other in a specific pathogen-free embryonated chicken egg (SPF-ECE) model.

**Materials and Methods:**

HPAIVs (H5N1 and H5N8) and NDVs [avirulent NDV [avNDV] and velogenic NDV [vNDV]) were inoculated into the allantois cavity of SPF-ECE with graded titers (2, 3, and 4 log_10_ EID50) at 24 and 48 h of incubation, followed by the collection of allantoic fluid. A quantitative reverse transcription real-time polymerase chain reaction was used to determine the viral RNA copies of both viruses.

**Results:**

Obvious interference was reported on the growth of NDVs when co-inoculated with AIVs. NDV RNA titers reduction ranged from <3 to 5 log_10_ to complete suppression, but slight interference with the growth of AIVs occurred. H5N1 RNA titers showed <1-2 log_10_ reduction when co-inoculated with vNDV compared with the H5N1 control. The interference impact of H5N8 was more powerful than that of H5N1, while vNDV showed more resistance for interference than the avNDV strain. On the other hand, interference of AIVs was not observed except when vNDV was inoculated before H5N1. The interfering impact was increased after 48 h of inoculation, whereas no titer of avNDV was detectable.

**Conclusion:**

AIV strains had a powerful effect on NDV growth, regardless of which infection occurred first.

## Introduction

Avian influenza (AI) and Newcastle disease (ND) are two major viral diseases that cause major losses to the poultry industry [1]. During the past decade, the poultry industry in Egypt was overwhelmed by the exposure to different AI virus (AIV) subtypes including the low pathogenic AIV (LPAIV) AI H9N2 and highly pathogenic AIVs (HPAIV) (HPAIV H5N1 and HPAIV H5N8) [2-4]. Meanwhile, ND continues to cause serious problems and high economic losses in the Egyptian poultry industry [5]. The genetic evolution of HPAIV in Egypt has been suggested to produce new clades 2.2.1.2 H5N1 and 2.3.4.4 H5N8; this raises the question of the effect of coinfection with other endemic viruses [6]. Mixed infection of both viruses caused major problems for the poultry industry due to severe economic losses and the wide range of infection that is accompanied by high morbidity and mortality as well as decreased egg production [7,8]. Several studies provide evidence for the high incidence of NDV-AIV mixed infections [8-10]. The prior growth of NDV may inhibit AIV growth resulting in false-negative AIV tests [11]. In a coinfection study, LPAIV had a negative impact on NDV growth when they were inoculated simultaneously or sequentially [12]. The previous infection of specific pathogen-free (SPF) chickens with virulent NDV strains can suppress HPAIV as a result of competition for cell surface receptors or competent cells required for replication [13]. The pre-infection of a host with one virus may affect the multiplication of a second virus, a phenomenon known as viral interference [14]. Veterinary authorities and poultry producers face the problem of mixed infections which are complicated by false diagnosis, the effect of one virus on another, and serious viral dissemination or a source of transmission [15]. Some research used chicken embryos as a model for studying mixed infection of AIV and NDV and their interference [16], where clinical and serological parameters were the predominant tools for studying the interference of mixed viral infection for poultry. Though, studies that quantitatively evaluate the degree of interference between both viruses are lacking [17]. On the other hand, studies on interference between AIV and NDV showed variable conclusions [15].

So, the significance of the current study become maximized as it discussed viral interference by evaluating AIV and NDV viral replication using Quantitative reverse transcription real-time polymerase chain reaction (qrRT-PCR).

This study aimed to evaluate the impact of viral interference by the dual infection of AIVs (H5N1-H5N8) and NDVs (avirulent NDV [avNDV]-velogenic NDV [vNDV]) in an SPF-embryonated chicken egg (SPF-ECE) model system using qrRT-PCR.

## Materials and Methods

### Ethical approval

This study does not require ethical approval as study was based on SPF-egg model (not living bird model).

### Virus strains

Four standard titrated viruses (of 10^6^ EID50 titer) were obtained from the repository of the National Laboratory for Veterinary Quality Control on Poultry Production (NLQP), Egypt [HPAIV-H5N1 (A/chicken/Egypt/173CAL/2017; HPAIV H5N8 (A/chicken/Egypt/CA35/2017; vNDV (NDV-GHB-328F-2016); and avNDV (NDV-CH-Behaira-Egypt-MR6-2012)]. GenBank accessions (for hemagglutinin [HA] gene for H5N1 and H5N8 AIVs and F gene for vNDV and avNDV NDVs) of the obtained strains are MG192004; MH762131; KX686728; and JX193771, respectively. Virus strains were 10-fold serially diluted to get the applied inoculum concentrations (10^2^, 10^3^, and 10^4^ EID50). The viral infectivity of each strain was determined by serial titration in 10-11-days-old embryonated eggs and was expressed as 50% of the egg infective dose (EID50/mL) using standard methods [18].

### SPF-ECE inoculation

SPF-ECEs were purchased from the Egyptian SPF Egg Production Farm (Nile SPF), Kom Oshiem, El-Fayoum Governorate, Egypt. ECEs were inoculated through the allantoic sac according to the OIE guidelines [19] and eggs were incubated at 37°C. Inoculated eggs were candled daily for 3-5 successive days. Bacteria-free allantoic fluid was aliquoted and stored at −80°C until tested.

### qrRT-PCR

Viral RNA extraction from the harvested allantoic fluids was performed using QIAamp viral RNA Mini kit (Qiagen, GmbH, Germany). The AIV-H5 HA gene and NDV matrix (M) gene were titrated in the purified RNA using standard QuantiNova real-time PCR kit (Qiagen, GmbH, Germany). qrRT-PCR was performed on a Mx3005P QPCR System (Agilent, California, USA). Samples with a Cq value ≤39 were considered positive. QrRT-PCR primers and probes for AIV-H5 gene [20] and NDV matrix gene [21] were supplied by Metabion (Germany).

### Experimental designs for reciprocal interference studies between AIVs and NDVs

The interference phenomenon was studied by two experiments, described below:

#### Experiment 1

Ten-days-old SPF-ECEs were sequentially infected with two viruses at equal multiplicities. In Table-1, a summary of the experimental design was provided. Four sets (sets 1-4) were divided individually into two groups (G1 and G2) of 15 eggs per group, each group was subdivided into three subgroups of varied titers 2, 3, and 4 log10 (five eggs per titer). The first set was designed for sequential inoculation of G1: Inoculation of AIV H5N1 followed by avNDV and G2: Inoculation of avNDV followed by H5N1 with 12 h in between the two inoculations. The set 2 design was the same as set 1, but with vNDV inoculation instead of avNDV. Sets 3 and 4 designs were the same as sets 1 and 2 designs, respectively (with inoculation of H5N8 instead of H5N1). Four positive control groups were designed for a single inoculation of each viral type, while sterile phosphate-buffered saline was injected instead of the second inoculum. One negative control group was also designed. After viral inoculations, ECEs were candled at 12 h intervals. Collection of allantoic fluid was done after 24 h.

**Table-1:** Experimental design of the study.

Sets	Set 1^a^		Set 2^a^		Set 3^a^		Set 4^a^		Positive control groups^c^				Neg control
						
Groups	G1	G2	G1	G2	G1	G2	G1	G2	H5N1	H5N8	vNDV	avNDV	Neg^d^
Each group included 15 ECEs (each group was divided into three subgroups of five eggs per titer), three different titers of each inoculated virus (2, 3, and 4 log_10_)													
First virus inoculation	H5N1^b^	avNDV^b^	H5N1^b^	vNDV^b^	H5N8^b^	avNDV^b^	H5N8^b^	vNDV^b^	H5N1	H5N8	vNDV	avNDV	PBS
Second virus inoculated after 12 h	avNDV^b^	H5N1^b^	vNDV^b^	H5N1^b^	avNDV^b^	H5N8^b^	vNDV^b^	H5N8^b^	PBS	PBS	PBS	PBS	PBS
Experiment 1 (allantoic fluid was collected after 24 h)													
QrRT-PCR	Sets 1-4 were tested for both H5 (H gene) and NDV (M gene)								H5	H5	NDV	NDV	H5 and NDV
Experiment 2 (allantoic fluid was collected after 48 h)													
QrRT-PCR	Sets 1-4 were tested for both H5 (H gene) and NDV (M gene)								H5	H5	NDV	NDV	H5 and NDV

a Each set included 30 eggs divided into two groups (15 eggs each).

b Each group was subdivided into three titers divisions, each titer was inoculated in five eggs. Then, equal titer of the second virus was inoculated in the same group.

c Four positive control groups (H5N1, H5N8, vNDV, and avNDV).

d Neg=Negative control, PBS=Phosphate-buffered saline, vNDV=Velogenic Newcastle disease virus, avNDV=Avirulent Newcastle disease virus, ECEs=Embryonated chicken egg, QrRT-PCR=Quantitative reverse transcription real-time polymerase chain reaction

#### Experiment 2

The same protocol was performed as experiment 1, but the collection of allantoic fluid was performed after 48 h. All the experiments were performed in duplicates.

### Statistical analysis

RNA copy titer of each virus was determined using qrRT-PCR. The degree of interference was estimated by comparing AIV or NDV yields from coinfected ECEs with those of the corresponding controls as measured independently by qrRT-PCR. Statistical variation between the experimental group and control group was determined by ANOVA test where p<0.05. This experiment was set up to investigate the effect of the first inoculated virus on the growth of a second inoculated virus in the ECE.

## Results

**Figure-1:**
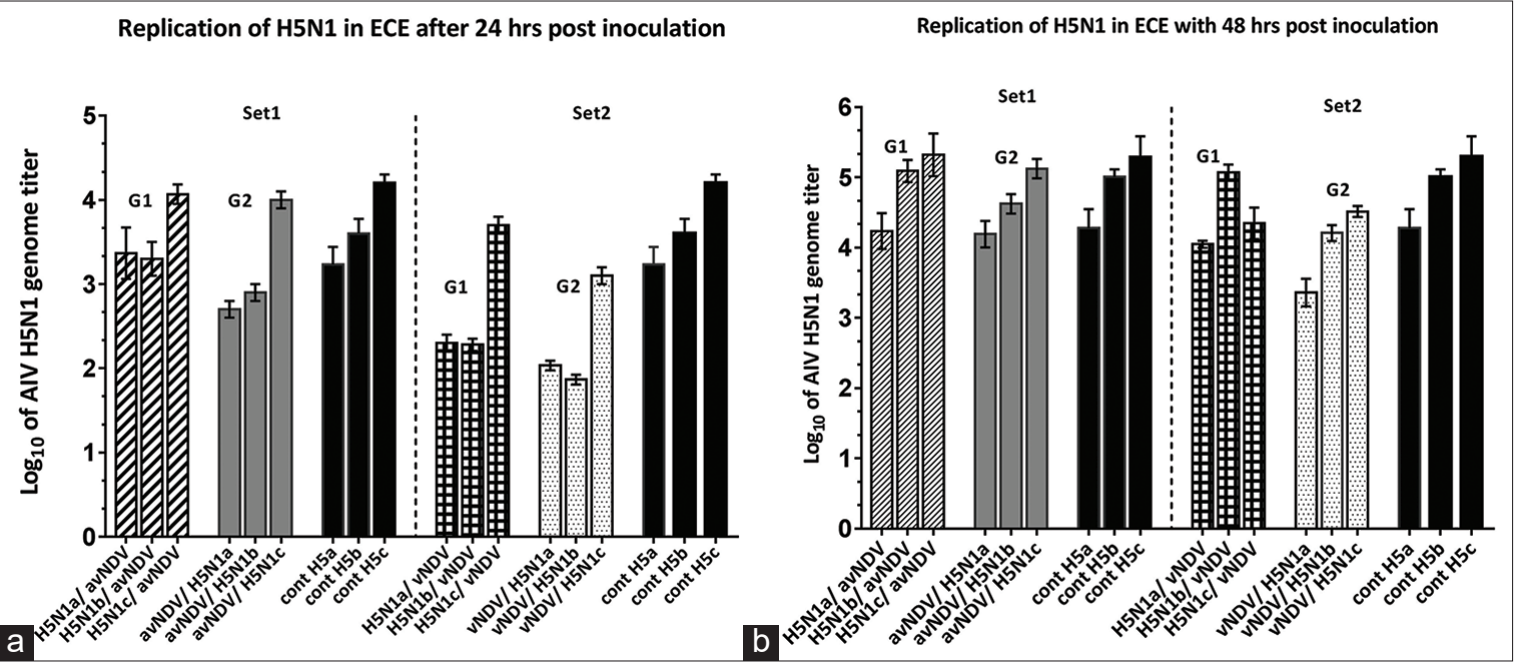
(a and b) The replication titers of H5N1 in sets 1 and 2. Set 1 [G1 (H5N1/avNDV), G2 (avNDV/H5N1)]. Set 2 [G1 (H5N1/vNDV), G2 (vNDV/H5N1)]. Black columns represent the positive control group (single H5N1 inoculation). a-c represent three different inoculated titers (2, 3, and 4 log_10_, respectively). Bars over the columns represent the error bars of standard deviation to the mean titers. p<0.05. a is related to 24 h incubation, b is related to 48 h incubation.

The results of experiments (1, 2) are shown in Figure-1, which represent the quantitative measure of H5N1 with reciprocal infection of avNDV and vNDV. AIV (H5N1) titers showed no reduction when ECEs were coinfected with avNDV. However, H5N1 RNA titers showed <1-2 log_10_ reduction when co-inoculated with vNDV compared with H5N1 control.

**Figure-2:**
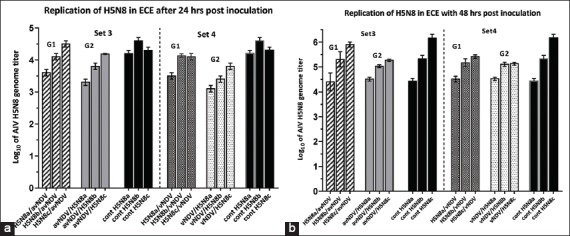
(a and b) The replication titers of H5N8 in sets 3 and 4. Set 3 [G1 (H5N8/avNDV), G2 (avNDV/H5N8)]. Set 4 [G1 (H5N8/vNDV), G2 (vNDV/H5N8)]. Black columns represent the positive control group (single H5N8 inoculation). a-c represent three different inoculated titers (2, 3, and 4 log_10_, respectively). Bars over the columns represent the error bars of standard deviation to the mean titers. p<0.05. a is related to 24 h incubation, b is related to 48 h incubation.

AIV (H5N8) titers showed no reduction when ECEs were coinfected with avNDV (Figure-2). However, H5N8 RNA titers showed one log_10_ reduction when co-inoculated with vNDV. The replication (irrespective of pre- or post-NDV infection) was compared to control single infection in ECE.

**Figure-3:**
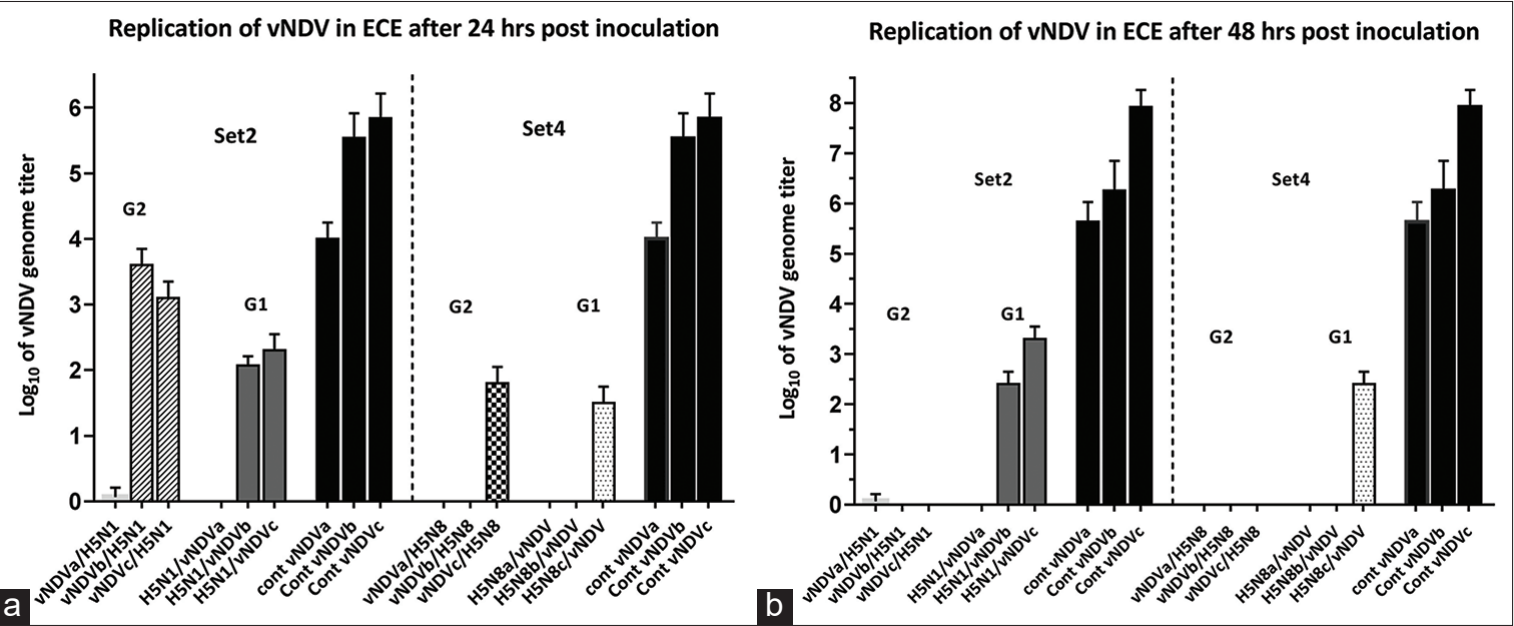
(a and b) The replication titers of vNDV in sets 2 and 4. Set 2 [G2 (vNDV/H5N1), G1 (H5N1/vNDV)]. Set 4 [G2 (vNDV/H5N8), G1 (H5N8/vNDV)]. Black columns represent the positive control group (single vNDV inoculation). a-c represent three different inoculated titers (2, 3, and 4 log_10_, respectively). Bars over the columns represent the error bars of standard deviation to the mean titers. p<0.05. a is related to 24 h incubation, b is related to 48 h incubation.

Virus yield of vNDV from dually infected ECE with H5N1 and H5N8 was significantly lower than those from singly infected ECE by a range of <3-5 log_10_ to complete inhibition (Figure-3a and b). The higher the level of infection with AIV virus, the greater the degree of interference observed. However, a prominent difference was found among H5N1 or H5N8 and virulent strain of NDV, where virulent NDV was more powerful in resisting interference induced by AIV (H5N1) (Figure-3a and b). Furthermore, findings showed that H5N8 was more powerful than H5N1 in inhibiting vNDV.

**Figure-4:**
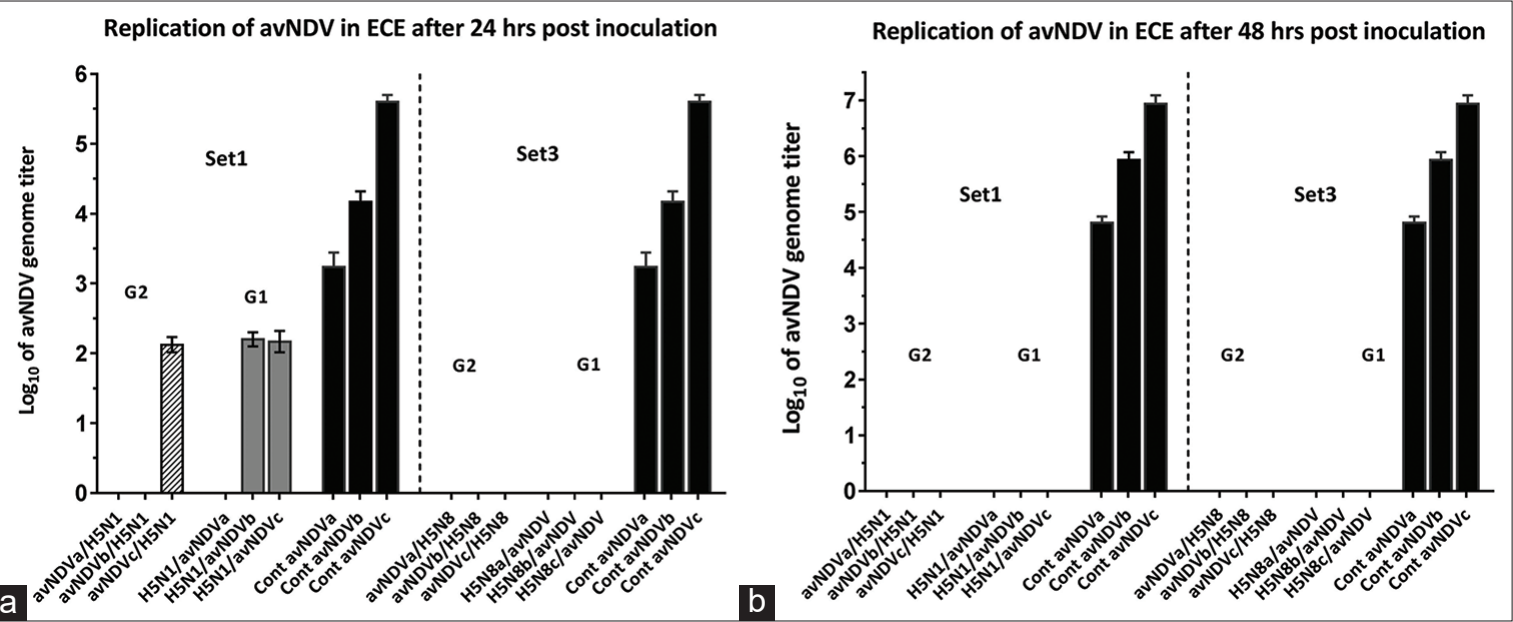
(a and b) The replication titers of avNDV in sets 1 and 3. Set 1 [G2 (avNDV/H5N1), G1 (H5N1/avNDV)]. Set 3 [G2 (avNDV/H5N8), G1 (H5N8/avNDV)]. Black columns represent the positive control group (single avNDV inoculation). a-c represent three different inoculated titers (2, 3, and 4 log_10_, respectively). Bars over the columns represent the error bars of standard deviation to the mean titers. p<0.05. a is related to 24 h incubation, b is related to 48 h incubation.

When equal titers of AIVs (H5N1 or H5N8) were sequentially inoculated, indications of interference on avNDV were observed (Figure-4). Only a high concentration of avNDV can slightly resist interference by H5N1 (p<0.05). No significant difference in reciprocal inoculation of NDV with AIV (H5N1 or H5N8) was recorded. The avNDV multiplicity interfered irrespective of which virus was inoculated first. It was obvious that the high titer of avNDV (4 log_10_) in infected ECE was relatively resistant to interference (Figure-4a). Another finding was that H5N8 was a more powerful agent than H5N1 in preventing the replication of NDV. As no viral yield of avNDV in different concentrations was detected (Figure-4b), whereas after 24 h post-inoculation of 3 and 4 log_10_ H5N1, a relative interference of avNDV was observed. However, after 48 h post-inoculation, complete interference of avNDV was recorded.

In view of the interfering effect of NDV (avNDV and vNDV) on AIV, NDV failed to inhibit the growth of AIV. The interference phenomenon was different after 48 h post-inoculation. The highest growth inhibition of NDV was observed when H5N8 was inoculated earlier than avNDV (Figure-4). Relative inhibition of vNDV with H5N8 and H5N1 was observed.

## Discussion

Viral interference is a common episode where one cell is infected with a virus that can inhibit the replication of secondary homologous or heterologous viruses [14]. In this study, interference of H5N1 and H5N8 HPAIV AIVs with avNDV and vNDV was quantitatively evaluated in ECE model using qrRT-PCR. Similar studies have shown that one virus is capable of inhibiting the growth of another [22,23]. The current study focused on the evaluation of the interference between AIV and NDV using qrRT-PCR to determine the degree of viral replication. Further, we studied the effect of some factors such as interfering doses, interfering intervals, and strain virulence.

AIV inhibited the growth of NDV, and the observed interference grade ranged from partial to complete interference according to titer and virulence of the viral strains. NDV downregulated AIV replication by one log when NDV was inoculated 24 or 48 h before AIV, allowing for an increase in replication. In contrast to a related study [17], the primary infection by NDV succeeded to inhibit to a lesser extent (<1-2 log_10_) the later AIV replication (Figures-1 and 2).

However, our result was corroborated by the previous research that supported the concept that pre-infection with NDV (either lentogenic or velogenic) can minimize later LPAIV or HPAIV AIVs replications [13].

In contrast, in the varied path of this study, the first inoculation of H5N8 could inhibit the replication of subsequent avNDV or vNDV inoculums for up to <2-3 log_10_ concentrations, but vNDV at high concentration like 4 log_10_ showed partial interference (Figure-3). This finding came in accordance with a study that reported that the previous infection of chickens with vNDV strains can reduce HPAIV replication. This interference depends on the viral titer, the virulence of NDV, and the timing of infections [24]. Furthermore, H5N1 could suppress the replication with vNDV or avNDV at 2 log_10_, but partial interference with high concentration 3 or 4 log_10_ was recorded only with vNDV though complete suppression was exerted by H5N1 on avNDV replication (Figures-3 and 4). Both outcomes suggest that vNDV is more potent in withstanding AIV interference than avNDV. In addition, the virulence of the viral strains was another important factor that affects interference in the current study. A previous study [25] reported the direct correlation between NDV strain virulence and the degree of replication.

A preceding study [26] also reported AIV-H9 interference due to NDV replication in ECEs. Another study was performed in SPF chickens that indicated that the previous infection of NDV can decrease the replication of HPAIV of H5N2 subtype [24]. A similar but more powerful interfering effect was reported by the previous study [11] where LPAIV-H9 was completely undetectable in cloacal swabs after Lasota vaccination.

Other studies reported the interfering effect exhibited by LPAIV-H7N2, where it significantly decreased the oral shedding of NDV in turkeys and chickens [13]. A resembling interfering effect was reported in a recent study where LPAIV-H9N2 delayed and decreased NDV shedding. They showed that the degree of viral interference is dose-dependent, which came in accordance with the findings in this study that the higher AIV inoculum dose reveals greater interfering effects on NDV replication [27].

Viruses exert their interfering action either through competing for cellular attachment as they reduce or even block the free cell receptors or by competing intracellularly for replication machinery [28].

The more probable AIV-NDV interference mechanism is the competition for cell receptor attachment as both viruses require sialic acid receptors either in the form of sialic acid-containing glycol conjugates for AIV [29] or gangliosides and N-glycoproteins for NDV [30].

Another mechanism for viral interference may be due to interferon induction due to primary viral infection that can suppress the replication of the secondary virus [31]. Such mechanisms were shown to be a possible mechanism for AIV-NDV interference [23]. This mechanism elucidates the strong inhibition of avNDV even when it was the primary infectious virus, as lentogenic NDV is a weak interferon inducer. The previous studies directly correlate the degree of interferon induction with the time interval between two infecting viruses [17,31]. This was taken into consideration in the current study, as there was a 12 h lag between the two viral inoculums to allow for maximum interferon activation. The current findings agree with the interference pattern exerted by interferon induction.

In the current study, we also report that H5N8 had a more powerful interfering force than H5N1. This complicates the case since in 2017, H5N8 HPAIV was in circulation in all Egyptian poultry sectors more than H5N1, so the chance of interference with NDV is currently higher than in the years before the entry of H5N8 in Egypt.

Although the interfering impact of avNDV was not obvious, it may lead to the misdiagnosis of AIV in coinfections with the Lasota strain due to lowering viral titers to an undetectable level that confound the correct diagnosis [10].

The study clarified the stronger capacity of vNDV compared with avNDV, in resisting the inhibition of AIV (inoculated later), also clarified the greater interfering capacity of H5N8 over that of H5N1, which was clearly demonstrated in this study.

## Conclusion

We summarize that AIV-NDV viral interference exists with a higher chance for AIV to inhibit NDV replication; however, the degree of interference may differ according to viral concentrations and strain virulence. Such episodes should be taken into consideration during field cases diagnosis to avoid false-negative results. The current study provides evidence that testing of interfering viruses during the molecular screening and viral isolation attempts of infected poultry flocks should be performed to identify the consequences of interference during coinfection. Finally, this study sheds light on the importance of planning for the diagnosis and control of avian disease among poultry sectors.

## Recommendations

The current search recommends studying the viral interference between AIVs and NDVs in living bird model.

## Authors’ Contributions

MAS designed this study and applied statistical analysis. AME performed molecular biology tests. AAN performed viral inoculations. All authors drafted, revised the manuscript, analyzed the data, and approved the final manuscript.
